# Coexisting Thyroglossal Duct Cyst with Papillary Thyroid Cancer: A Case Report and Literature Review

**DOI:** 10.1155/2021/6111308

**Published:** 2021-12-26

**Authors:** Abdullah A. Alarfaj, Ahmed Zekri, Ibrahim Alyaeesh, Ahmed Alomairin, Abdulrahman Al Naim

**Affiliations:** Department of Surgery King Faisal University, Alahsa, Saudi Arabia

## Abstract

Thyroglossal duct cysts (TGDCs) are common developmental anomalies in which the thyroglossal duct is not obliterated. Coexisting papillary thyroid cancer and TGDC are uncommon and should be investigated thoroughly to rule out TGDC carcinoma. We report a rare case of coexisting papillary thyroid cancer and TGDC in a 48-year-old man, who presented with a history of recurrent mild painful midline neck swelling, and ultrasound (US) revealed a TGDC that was subsequently managed conservatively. On follow-up after 1.6 years, a thyroid US and a fine-needle aspiration (FNA) biopsy were performed, which showed malignant papillary thyroid carcinoma. Total thyroidectomy, the Sistrunk procedure, and central neck dissection were implemented. After three days, the patient was discharged on 150 mg of levothyroxine. Follow-up was unremarkable with no complications. The authors would like to stress the importance of regular TGDC and thyroid gland follow-ups for early detection and diagnosis of thyroid malignancy via clinical examination and US.

## 1. Introduction 

Thyroglossal duct cysts (TGDCs) or sinuses develop when the thyroglossal duct is not obliterated. The thyroglossal duct, which is an embryological remnant of the thyroid gland descent pathway, connects the thyroid gland to the base of the tongue and extends from the foramen cecum to the midtrachea. The thyroglossal duct usually involutes at 7–10 weeks' gestation [1–4]. TGDCs affect approximately 7% of the worldwide population, with around 1% reportedly hosting malignant tumors, most commonly papillary-type thyroid cancer (>90%) [5]. Most TGDC malignancies are suspected due to the presence of certain examination and imaging features, such as a rapidly growing, solid mass within a TGDC. Using fine-needle aspiration (FNA) biopsies is controversial because of their low accuracy for diagnosing TGDC malignancies [5–8]. Hence, it is still difficult to preoperatively diagnose TGDCs with malignancies because the preoperative workup results are almost identical to the findings for TGDCs without malignancies [5, 9, 10]. The co-occurrence of thyroid carcinoma with TGDC carcinoma has a relatively high incidence, being reported in 25–56% of cases [11]. Thyroid cancer should be suspected in the presence of a thyroid nodule, which accounts for 7–15% of cases. However, there is a 90% chance of this nodule representing a differentiated thyroid cancer, which includes either papillary or follicular cancer [12]. The best management of TGDCs with papillary carcinomas is the Sistrunk procedure, with or without thyroidectomy based on thyroid findings [13]. However, there are no clear guidelines for managing thyroid lobe carcinomas, which can also occur in association with TGDCs [14, 15].

Along with a review of the current literature, this paper will present a rare case of TGDC co-occurring with left lobe thyroid papillary carcinoma.

## 2. Case Report

**Figure 1:**
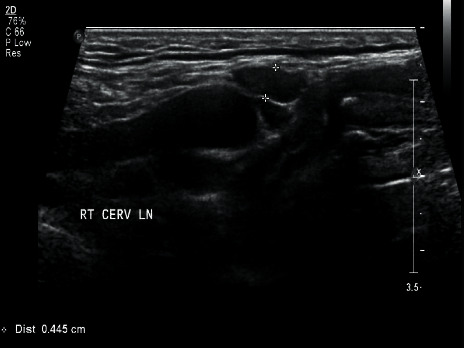
Preoperative US of TGDC.

**Figure 2:**
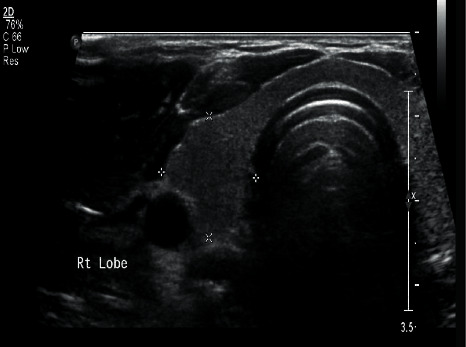
Preoperative US of thyroid nodule.

**Figure 3:**
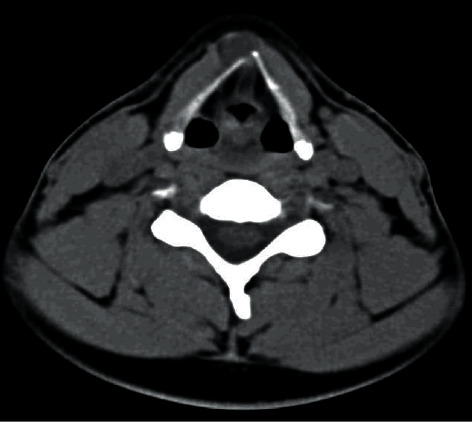
Axial cross section of a soft-tissue window CT image of the TGDC.

**Figure 4:**
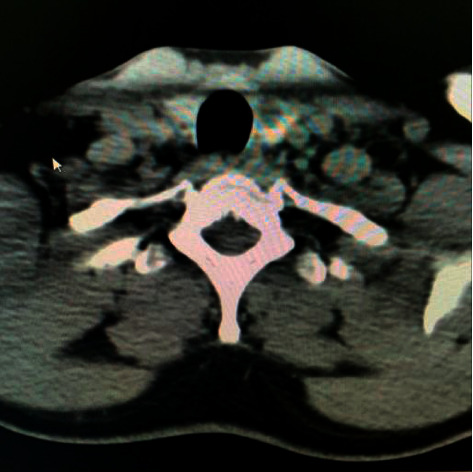
Axial cross section of a soft-tissue window CT image of the left thyroid nodule.

**Figure 5:**
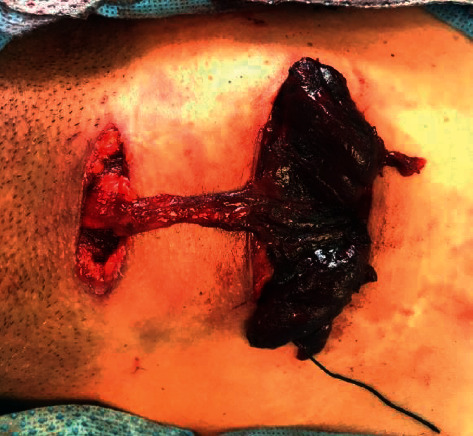
Intraoperative picture of the surgical incisions and specimens (TGDC and thyroid gland).

A 48-year-old, medically free man presented to the Ear, Nose, and Throat (ENT) clinic at King Abdulaziz National Guard Hospital in Al Ahsa, Saudi Arabia. He had been referred from a family medicine clinic with a history of recurrent, mildly painful midline neck swelling, which had been occurring for one and a half months before presentation. The patient exhibited no dyspnea, dysphagia, or signs of hypo- or hyperthyroidism. He also had no significant past medical, family, surgical, or nutritional history. In the ENT clinic, the physical examination was unremarkable, except for a small, painless, midline neck mass at the level of the hyoid bone. Flexible nasolaryngoscopy revealed bilateral vocal cord movement with no noticeable lesion. Ultrasound (US) was conducted, revealing fluctuant hypoechoic cystic structures, the walls of which were thin and without internal vascularity. A TGDC diagnosis was determined, and the patient was managed conservatively. However, due to the remarkably high rate of malignancy when TGDCs are present in elderly patients, a regular ENT and US follow-up was scheduled every six months. After 1.6 years, follow-up neck US showed a hypoechoic lesion in the midline and slightly to the right side of the neck, measuring 5 × 8 mm at the level of the hyoid bone. An isoechoic nodule, measuring 9 × 8 mm, was also incidentally discovered in the left thyroid lobe. The isthmus was normal in thickness and echogenicity, and there was no cervical lymphadenopathy (Figures 1 and 2). Then, a US-guided FNA biopsy from the left thyroid gland nodule was conducted using the complete aseptic technique; one needle pass was performed. The FNA biopsy showed malignant papillary thyroid carcinoma, categorized as Level VI by the Bethesda system. Computed tomography (CT) showed a cystic lesion in the infrahyoid location in the substance of the strap muscle abutting the thyroid cartilage, slightly off midline towards the right side, suggestive of a TGDC. A small nodule was also seen in the left lobe of the thyroid (Figures 3 and 4). The patient was given the options of hemithyroidectomy or total thyroidectomy, and he chose the second one. Total thyroidectomy, the Sistrunk procedure, and central neck dissection were implemented (Figure 5). The histopathology of the thyroid nodule specimen showed groups of follicular epithelial cells, arranged in a papillary structure and in loosely cohesive sheets, with several intranuclear inclusions and nuclear grooves present, as well as gummy colloid. The patient, therefore, was diagnosed with classical papillary thyroid carcinoma (T1bN0aM0), measuring 1.2 cm in the left lobe, and a TGDC, which was free of papillary carcinoma. Lymph nodes showed no metastasis. Postoperatively, the patient was doing fine with no postop complications, and the surgical wound was clean. On day three, the patient was discharged on 150 mg of levothyroxine. Follow-up with clinical examination, US, CT, and thyroid function tests was unremarkable, and the incision healed normally with no complications.

## 3. Discussion

\The thyroid gland arises from the foramen cecum, descends to the peritracheal space, and remains there. TGDCs can form if there is an anomaly in the descent of the thyroid gland in its tract during development [1]. The patient discussed here was being followed up for an infected TGDC, which is a common complication that makes patients complain of pain, tenderness, and swelling of the anterior part of the neck [16]. Carcinoma is rare but is reported to occur in around 1% of all TGDC cases, with a mean age of 39.5 years. Most TGDC carcinomas present as an asymptomatic neck mass, which necessitates regular TGDC follow-up clinically and via imaging techniques (US or CT scan) [14, 16].

Bethesda classification is employed to report and interpret the findings of thyroid cytology. The present FNA biopsy report for this patient's thyroid nodule showed Bethesda Level VI, which is malignant most of the time [17]. The next step, therefore, was to conduct neck and chest CT, as this can be very helpful in preoperatively evaluating the magnitude of the spread of malignancy and neck lymph node involvement [18].

In conducting a literature review, the present authors have not found any clinical guidelines for managing the thyroid lobe carcinoma that occurs in association with TGDCs [3]. Cases have been reported in which a benign TGDC and thyroid carcinoma simultaneously occurred. In two cases reported by Adele Bahar in 2020, Sistrunk's procedure was implemented in both cases, combined with total thyroidectomy in one instance and near-total thyroidectomy in the other. In the third case, reported by Jaromfr Astl in 2003, total thyroidectomy and the modified Schlange procedure were combined to remove the TGDC. Total or near-total thyroidectomy in combination with complete removal of the TGDC, whether with Sistrunk's or the modified Schlange procedure, was justified in the previous cases, as both pieces of research believe that not all TGDC carcinomas primarily originate from the TGDC and may, instead, be the result of metastasis from thyroid cancer. Therefore, similar malignant features can be found in the postoperative histopathology of a thyroid nodule and a TGDC [15, 19].

In the present patient, the Sistrunk procedure was implemented for the TGDC, along with classical total thyroidectomy and central neck dissection for papillary thyroid carcinoma, as there is a high incidence of the co-occurrence of thyroid carcinoma and TGDC carcinoma (25–56%) [11]. The authors performed these procedures using two incisions: one in the inferior part of the neck and the other in the upper part of the neck to access the TGDC as it was in a high location.

## 4. Conclusion

The authors would like to stress the importance of regular TGDC and thyroid gland follow-ups for early detection and diagnosis of thyroid malignancies using clinical examination and US. They also recommend regular follow-ups for a TGDC, even if it is stable and asymptomatic, as this could be a path to diagnosing other thyroidal or TGDC pathologies.

## Abbreviations

TGDC: Thyroglossal duct cyst
US: Ultrasound
CT: Computed tomography
FNA: Fine-needle aspiration.
